# Megakaryocyte lineage development is controlled by modulation of protein acetylation

**DOI:** 10.1371/journal.pone.0196400

**Published:** 2018-04-26

**Authors:** Marije Bartels, Anita Govers, Roel Polak, Stephin Vervoort, Ruben van Boxtel, Cornelieke Pals, Marc Bierings, Wouter van Solinge, Toine Egberts, Edward Nieuwenhuis, Michal Mokry, Paul James Coffer

**Affiliations:** 1 Center for Molecular Medicine and Regenerative Medicine Center, University Medical Center Utrecht, Utrecht, The Netherlands; 2 Division of Pediatrics, University Medical Center Utrecht, Utrecht, the Netherlands; 3 Department of Hematology, Erasmus Medical Center, Rotterdam, The Netherlands; 4 Department of Developmental Biology and Stem Cell Research, Hubrecht Institute, Utrecht, the Netherlands; 5 Department of Clinical Chemistry and Haematology, University Medical Center Utrecht, Utrecht, the Netherlands; 6 Department of Clinical Pharmacy, University Medical Center Utrecht, Utrecht, The Netherlands; European Institute of Oncology, ITALY

## Abstract

Treatment with lysine deacetylase inhibitors (KDACi) for haematological malignancies, is accompanied by haematological side effects including thrombocytopenia, suggesting that modulation of protein acetylation affects normal myeloid development, and specifically megakaryocyte development. In the current study, utilising ex-*vivo* differentiation of human CD34+ haematopoietic progenitor cells, we investigated the effects of two functionally distinct KDACi, valproic acid (VPA), and nicotinamide (NAM), on megakaryocyte differentiation, and lineage choice decisions. Treatment with VPA increased the number of megakaryocyte/erythroid progenitors (MEP), accompanied by inhibition of megakaryocyte differentiation, whereas treatment with NAM accelerated megakaryocyte development, and stimulated polyploidisation. Treatment with both KDACi resulted in no significant effects on erythrocyte differentiation, suggesting that the effects of KDACi primarily affect megakaryocyte lineage development. H3K27Ac ChIP-sequencing analysis revealed that genes involved in myeloid development, as well as megakaryocyte/erythroid (ME)-lineage differentiation are uniquely modulated by specific KDACi treatment. Taken together, our data reveal distinct effects of specific KDACi on megakaryocyte development, and ME-lineage decisions, which can be partially explained by direct effects on promoter acetylation of genes involved in myeloid differentiation.

## Introduction

Transcriptional regulation of the megakaryocyte/erythroid (ME) lineage is regulated by a relatively small number of master regulators. Differentiation of the common myeloid progenitor (CMP) towards the ME-lineage in humans is mainly regulated by GATA-1, the key-regulator of both megakaryocyte, and erythrocyte development [1–3]. Progression from the megakaryocyte/erythroid progenitor (MEP) towards megakaryocyte differentiation involves the activity of Runt-related transcription factor 1 (RUNX1, or AML1), LIM domain only 2 (LMO2), nuclear factor, erythroid-derived 2 (NF-E2), and Friend leukaemia integration 1 (Fli-1), which regulates the expression of late stage megakaryocyte markers [4–7]. Megakaryocyte maturation coincides with endomitosis, resulting in large, polyploid cells, the hallmark of megakaryocyte development. While previous studies have suggested a role for genes involved in cell cycle regulation and cytokinesis, including survivin and Aurora B, the regulation of polyploidisation remains unclear [8–11].

In addition to transcriptional regulation of normal and aberrant myeloid differentiation, the role of epigenetic regulatory networks, and post-translational modifications has been identified in recent years [12–16]. This has resulted in an increased use of chromatin modulating drugs, including lysine deacetylase inhibitors (KDACi) for the treatment of haematological malignancies [17–19]. KDACi inhibit deacetylation of histone and non-histone protein substrates, suggesting that the regulation of protein acetylation plays an important role in the cellular effects of KDACi in malignant cells. Since KDACi are particularly effective in myeloid disorders, including myelodysplastic syndrome (MDS), and acute myeloid leukemia (AML), this raises questions as to the role of protein acetylation in normal myeloid development [20–27].

Results of phase I/II clinical trials with KDACi suggest no unfavorable effects on the normal haematopoietic progenitor cell (HPC) compartment, however both hypergranulocytosis, and prolonged thrombocytopenia have been described [20, 28–31]. Previous studies in normal HPCs have demonstrated that valproic acid (VPA), a class I/IIa KDACi, stimulates the expansion of myeloid progenitor cells at the expense of myeloid differentiation [32–35]. With respect to ME-lineage development, a recent microarray based study in myeloid cell lines and CD34+ cells, suggested inhibitory effects of VPA treatment on erythropoiesis, illustrated by down-regulation of GATA1/FOG1 expression [36]. Others have suggested a stimulatory effect of VPA on ME-lineage development, illustrated by increased numbers of megakaryocyte, and erythroid precursors [37]. Moreover, treatment with VPA, a commonly used anti-epileptic drug, is associated with a large variety of haematological side effects, including thrombocytopenia, in patients with no previous haematological condition. Next to the effects of ME-lineage development, thrombocytopenia is possibly due to increased platelet clearance [38–41]. Previous studies utilising the class III KDAC/sirtuin inhibitor nicotinamide (NAM) have suggested a role for SIRT1 in human megakaryocyte maturation, involving the regulation of polyploidisation, yet the underlying molecular mechanisms remain unclear [42, 43].

In this study, we compared the effects of VPA treatment with NAM treatment on human ME-lineage development, and further progression into the megakaryocytic lineage. Our data demonstrate for the first time that KDAC and SIRT inhibition differentially modulates the expansion and differentiation of MEP. Treatment with VPA increases the MEP compartment, yet inhibits megakaryocyte development, while erythroid development is normal. NAM treatment stimulates megakaryocyte differentiation at the expense of proliferation, while the effects on erythroid development resemble the effects of VPA treatment. Utilising a histone 3 lysine 27 acetylation (H3K27ac) chromatin immunoprecipitation- (ChIP) sequencing approach, we identified key regulatory genes implicated in myeloid progenitor function, and ME-lineage differentiation, directly regulated by VPA and NAM treatment. Taken together, our study provides novel insights into the effects of KDACi on ME-lineage development, and increases our knowledge of the role of HDAC and sirtuins in normal human haematopoiesis.

## Materials and methods

### UPOD analysis of patient data

Data were obtained from the Utrecht Patient Oriented Database (UPOD). The content of UPOD and its setting have been described in detail elsewhere [44, 45]. From UPOD, all outpatients, both adults and children, were identified who were treated with VPA (N = 217) and had at least one haematological blood test together with a VPA plasma level test on the same day from January 2005 until December 2009. For all patients, total thrombocyte counts were determined.

### Isolation and culture of human CD34+ cells

CD34+ cells were isolated from human umbilical cord blood as previously described (32). CD34+ (3.0 x 10^5^) cells were cultured in Stemspan Serum Free Expansion Medium (SFEM) (Stemcell technologies SARL, Grenoble, France). Cells were differentiated towards megakaryocytes for 11 days upon addition of stem cell factor (SCF) (50 ng/mL), thrombopoietin (TPO) (20 ng/mL), and interleukin 6 (IL-6) (10 ng/mL). Cells were differentiated towards erythrocytes for 11 days upon addition of SCF (50 ng/mL) interleukin-3 (IL-3) (0.1 nmol/L), and eryhtropoietin (EPO) (5IE/mL). Every 3 days, cells were counted with trypan blue, and fresh medium was added to a density of 5.0 x 10^5^ cells/mL for megakaryocytes, and 2.0 x 10^5^ for erythrocytes. After 7 days of differentiation only TPO and IL-6 were added to the megakaryocyte cultures. The KDAC inhibitors trichostatin A (TSA), sodium butyrate (SB) valproic acid (VPA) (Alexis Chemicals, Lausen, Switzerland) and SIRT inhibitor nicotinamide (NAM, Sigma-Aldrich Chemie B.V., Zwijndrecht, the Netherlands) were added to the fresh medium every 3 days. Umbilical cord blood was collected after written informed consent was provided according to the Declaration of Helsinki. The use of umbilical cord blood for this study was approved by the ethics committee of the University Medical Center Utrecht.

### Measurement of apoptosis

Cells were isolated at day 4, 7 and 11, and the percentage of apoptotic cells was analysed by FACS as previously described [32]. Due to normal variation between donors, we expressed the effects of VPA and NAM treatment on apoptosis related to apoptosis in the controls.

### FACS analysis of megakaryocyte and erythrocyte differentiation

Mature megakaryocytes can be characterised by cell surface expression of integrin β3 (CD61), and glycoprotein 1B (CD42b), and by polyploidy (DNA content >4N). Mature erythrocytes can be characterized by transferrin receptor protein 1 (CD71), and glycophorin A (CD235a) expression. Megakaryocyte precursor cells were isolated at day 7, and 11 of differentiation, washed with PBS, followed by incubation phyoerythrin (PE)-labeled anti-CD61 antibody, and a fluorescein isothiocyanate (FITC)-labeled anti-CD42b antibody (Becton Dickinson, Alphen a/d Rijn, the Netherlands) in PBS/5% FCS on ice, for 30 minutes in the dark. Next, cells were washed in PBS, resuspended in PBS/5% FCS, and analysed by FACS. Erythrocyte precursor cells were isolated after 11 days, and incubated with PE-labeled anti-CD71 antibody, and FITC-labeled anti-CD235a antibody, and prepared for FACS analysis as described above. Isotype antibody staining was used for gating. Analysis of polyploidy was performed after 7, and 11 days of differentiation. Cells were stained with FITC-labeled conjugated CD42b antibody as described before. After that, cells were fixed and permeabilised in ice-cold ethanol for 30 minutes on ice. Cells were washed with PBS, resuspended in PBS/5mM EDTA and incubated with 40μg/mL RNAse for 30 minutes at RT. Subsequently the cellular DNA content was stained with 0.1 mg/mL propidium iodide (Bender MedSystems, Vienna, Austria) for 5 minutes and analysed in the CD42b-positive cell population by FACS (FACS Canto, Becton Dickinson, Alphen a/d Rijn, The Netherlands). Due to normal variation between donors, we expressed the effects of VPA and NAM treatment on MK differentiation related to MK differentiation in the controls.

### Histochemical staining of haematopoietic cells

May-Grunwald Giemsa staining was used to analyse megakaryocyte differentiation, and erythrocyte differentiation. Cytospins were prepared from 5.0 x 10^4^ differentiating megakaryocytes and were fixed in methanol for 3 minutes. After fixation, cytospins were stained in a 50% eosin methylene blue solution according to May-Grunwald (Sigma Aldrich, Seelze, Germany) for 15 minutes, rinsed in water for 5 seconds, and nuclei were counterstained with 10% Giemsa solution (Merck kGaA, Darmstadt, Germany) for 20 minutes. Megakaryocyte maturation can be characterised by polyploidisation. Erythrocyte differentiation can be characterised by enucleation to produce reticulocytes. Micrographs were acquired with an Axiostar plus microscope (Carl Zeiss, Sliedrecht, the Netherlands) fitted with an 100x/1.3 NA EC Plan Neofluor oil objective using Immersol 518F oil (Carl Zeiss), a Canon Powershot G5 camera (Canon Nederland, Hoofddorp, the Netherlands), and Canon Zoombrowser EX image acquisition software. Photoshop CS5 was used for image processing (Adobe Systems Benelux, Amsterdam, The Netherlands).

### Myeloid progenitor staining

Haematopoietic progenitor populations were characterised as described by Manz *et al*. [46]. Briefly, CD34+ cells were isolated and cultured to induce megakaryocyte differentiation as described above for 4 days. Cells were subsequently washed and resuspended in PBS/5% FCS (Hyclone, South Logan, Utah, USA) and incubated for 30 min on ice with a mixture of antibodies (all from Becton Dickinson, Alphen a/d Rijn, The Netherlands). Lineage markers included CD2, CD3, CD4, CD7, CD8, CD14, and CD235a and myeloid progenitors are negative for these markers. The lineage negative (Lin-), CD34+, and CD38- population consists of haematopoietic stem cells (HSC). Lin-, CD34+, CD38+, CD123+, and CD45RA^-^ cells are common myeloid progenitors (CMP), whereas Lin-, CD34+, CD38+, CD123+, and CD45RA+ cells are granulocyte-macrophage progenitors (GMP). The Lin- CD34+, CD38+ CD123- and CD45RA- cell population contains the megakaryocyte-erythroid progenitors (MEP). Cell populations containing HSC, CMP, GMP and MEP were characterized by FACS analysis (FACS Canto, Becton Dickinson, Alphen a/d Rijn, The Netherlands). Isotype antibody staining was used to ensure gating of the correct population.

### Chromatin immunoprecipitation (ChIP) and sequencing

CD34+ cells were differentiated towards megakaryocytes for 4 days, followed by overnight treatment with VPA, or NAM. Lysates were prepared, and ChIP was performed as described previously [47] utilising an anti-acetylated H3K27 antibody (ab4729, Abcam, Cambridge, MA, USA). Next, chromatin was sheared, end-repaired, followed by ligation of sequencing adaptors, amplification of the library by ligation–mediated PCR (LMPCR). After LMPCR, the library was purified, checked for the proper size range, and the absence of adaptor dimers on a 2% agarose gel, followed by sequencing on the SOLiD/AB sequencer (Applied Biosystems Life Technologies, Carlsbad, CA, USA). Sequencing reads were mapped against the reference genome (hg19,NCBI3) using the BWA package (-c–l 25 –k 2 –n 10) [48]. Non-uniquely placed reads were discarded. Cisgenome v2.0 software package [49] was used for the peak calling from the ChIP-seq data with settings–e 50 –maxgap and further analysis. Cisgenome 2 was used with settings: -e 50, -maxgap 200 and -minlen 200. A combination of Cisgenome functions, custom PERL and R scripts was used for additional data analysis. Data were normalised for the total amount of acetylation.

### Quantification of RNA expression

CD34+ cells were differentiated towards megakaryocytes for 4 days, followed by overnight treatment with VPA, or NAM. mRNA was extracted using the RNeasy Isolation Kit (Qiagen, Copenhagen Denmark). According to the manufactures protocol for single-stranded cDNA synthesis, 500 ng of total RNA was reverse transcribed using iScript cDNA synthesis kit (BIO-Rad, Hercules, CA). cDNA samples were amplified using SYBR green supermix (BIO-Rad), in a MyiQ single-color real time PCR detection system (BIO-Rad) according to the manufacturers’ protocol. To quantify the data, the comparative Ct method was used. Relative quantity was defined as 2^− ΔΔCt^ and *ß2 microglobulin* was used as reference gene. The sequences of the primers are listed in Table 1.

**Table 1:** qPCR primers.

Gene	Forward primer	Reverse primer
GATA 1	GCCCAAGAAGCGCCTGATTGT	TTCCGCATGGTCAGTGGCCG
LMO2	GGACCCTTCAGAGGAACCAG	TAGCGGTCCCCAATGTTCTG
RUNX1	ACAGCCATGAGGGTCAGCCCA	GGTGCTGTGTCTTCCTCCTGCAT
RUNX2	GCCTTCAAGGTGGTAGCCC	CGTTACCCGCCATGACAGTA

### Statistics

The correlation between VPA plasma concentration and the absolute number of platelets was evaluated using linear regression analysis. Data analysis was performed using SPSS 17.0 (SPSS Inc. Chigago, IL). For analysis of the other experiments, a one-way ANOVA analysis was followed by a Dunnett multiple comparison test to compare the differences between the control and KDACi-treated cells, or a Bonferroni multiple comparison test to compare all treatment conditions. A p-value of 0.05 or less was considered significant: * p<0.05, ** p<0.01.

## Results

### KDAC inhibitors differentially affect terminal megakaryocyte differentiation

**Fig 1:**
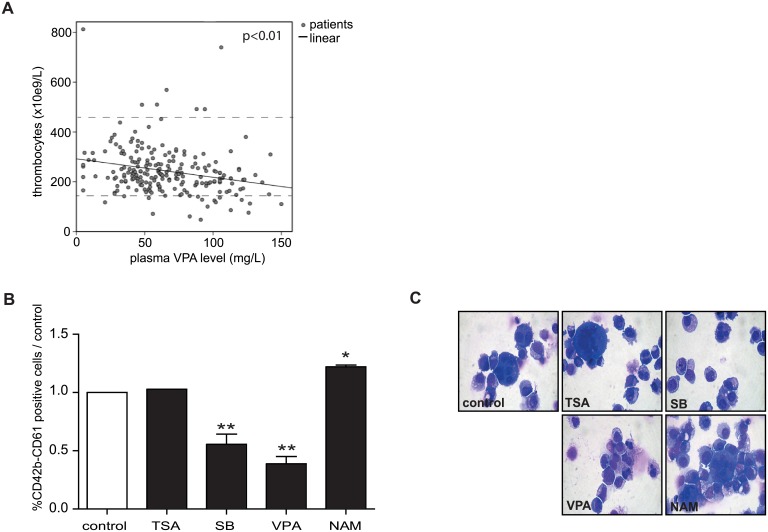
KDACi treatment differentially modulates megakaryocyte development. (A) From the peripheral blood the absolute number of platelets was analysed in patients treated with VPA (n = 217) together with plasma VPA concentration, measured at the same day. Data represent linear regression analysis of the absolute number of platelets (dependent variable), and plasma VPA concentration (independent variable). (B) UCB-derived CD34+ cells were differentiated towards megakaryocytes in the absence or presence of 10nM TSA, 250μM SB, 200μM VPA, or 1mM NAM for 11 days. Differentiation was determined based on the surface expression of CD61 and CD42b (percentage of double positive cells compared to the control), and (C) cytospin analysis. Data are representative for 3 independent experiments. Error bars represent SEM, * p<0.05, ** p< 0.01.

The unique process of megakaryocyte development is characterised by lineage progression from a bipotent progenitor (MEP), followed by endomitosis, resulting in highly specialised, polyploid cells (S1 Fig). The effects of treatment with valproic (VPA) acid and other class I/II KDAC inhibitors on megakaryocyte differentiation and thrombocyte formation are still under debate [36, 37, 50]. To initially evaluate the potential effect of KDAC inhibition on megakaryocyte development, we first evaluated the correlation between VPA plasma concentration and thrombocyte numbers in the peripheral blood by utilising data from the Utrecht Patient Oriented Database (UPOD). We analysed 217 non-haematological outpatients who were treated with VPA and had at least one haematological blood test together with a VPA plasma level. Our data demonstrate that increasing concentrations of VPA are significantly, inversely correlated with absolute thrombocyte numbers (Fig 1A).

Next, to determine whether we could recapitulate these observations *in vitro*, we differentiated human umbilical cord blood (UCB)-derived CD34+ hematopoietic progenitor cells towards megakaryocytes in the absence or presence of the pan KDACi TSA, two different class I/II KDACi (VPA or SB), and compared this with treatment with the class III (sirtuin) inhibitor nicotinamide (NAM). Megakaryocyte differentiation was determined based on integrin β3 (CD61), and glycoprotein 1B (CD42b) expression, and cytospin analysis of megakaryocyte morphology. While we observed no clear effects of TSA on megakaryocyte differentiation, VPA and SB treatment resulted in a significantly decreased CD61/CD42b expression (Fig 1B) and morphologically small immature megakaryocyte precursor cells (Fig 1C). In contrast, treatment with nicotinamide resulted in a significant increase in CD61/CD42b expression, and morphologically more mature and polyploid megakaryocytes (Fig 1B and 1C). Together these data suggest that class I/II KDACi inhibit, while SIRT inhibition stimulates megakaryocyte differentiation.

### VPA and NAM have differential effects on megakaryocyte differentiation

**Fig 2:**
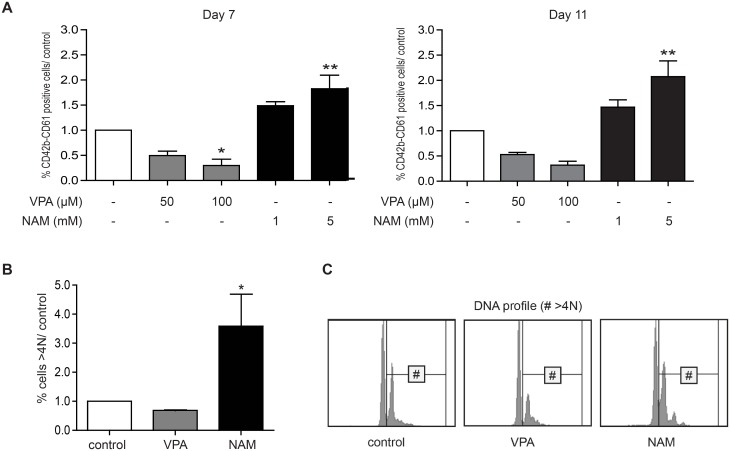
VPA and NAM have opposing effects on megakaryocyte differentiation. UCB-derived CD34+ cells were differentiated towards megakaryocytes for 11 days in the absence or presence of 100–200μM VPA, or 1-5mM NAM. (A) At day 7 and 11 of differentiation surface expression of CD61 and CD42b was analysed by FACS. Data represent the percentage of double positive cells, compared to the control. (B) At day 11 of differentiation, cellular DNA content was analysed by FACS. Data represent the percentage of polyploid cells (>4N), compared to the control within the CD42b positive population (left panel), and FACS histogram plots of DNA profile (right panel). Data are representative for 3 independent experiments. Error bars represent SEM, * p<0.05, ** p< 0.01.

Since megakaryocyte differentiation was most dramatically affected by treatment with VPA and NAM, we investigated the effects of these compounds on megakaryocyte development in more detail. UCB derived CD34+ cells were differentiated towards megakaryocytes for 11 days and the expression of CD61 and CD42b was analysed during differentiation. Treatment with 100μM VPA resulted in a significant decrease in CD61/CD42b expression at day 7 of differentiation, while treatment with NAM 5mM significantly increased the expression of these markers (Fig 2A). Since megakaryocyte differentiation, and more importantly megakaryocyte function, is characterised by polyploidisation of megakaryocyte precursors, we also analysed the effects of VPA and NAM treatment on the percentage of polyploid cells. In agreement with the effects of these compounds on surface marker expression and megakaryocyte morphology, NAM treatment resulted in a significant increase in the percentage of polyploid cells. A small decrease in the percentage of polyploid cells upon treatment with VPA was observed, suggesting that the effects of VPA on megakaryocyte development predominantly involved maturation, while polyploidisation is relatively unaffected (Fig 2B).

### VPA and NAM treatment inhibits the expansion of megakaryocyte precursors

**Fig 3:**
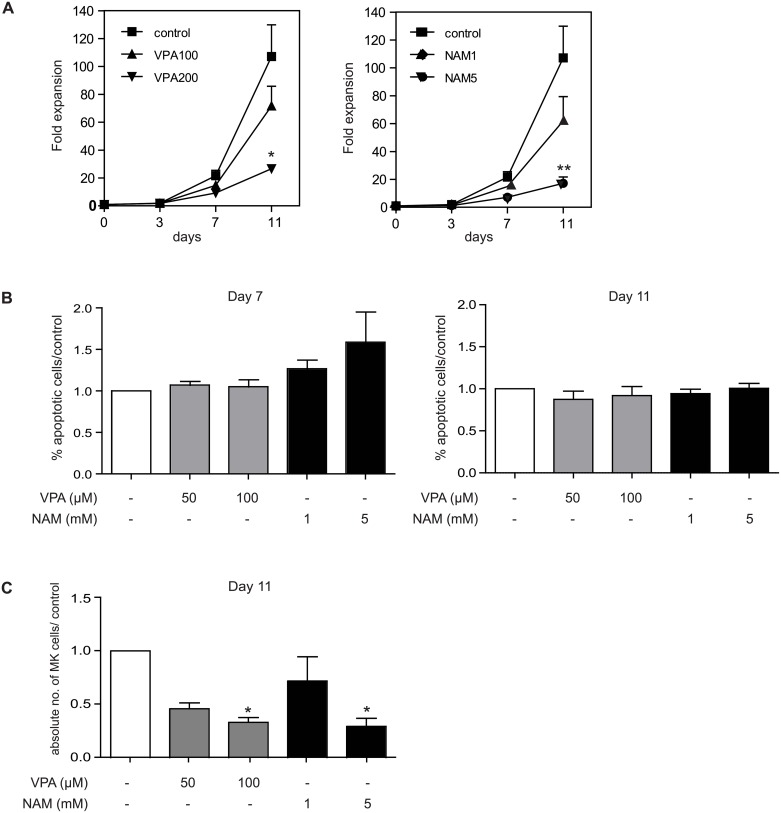
VPA and NAM treatment inhibits megakaryocyte progenitor proliferation. UCB-derived CD34+ cells were differentiated towards megakaryocytes for 11 days in the absence or presence of 100–200μM VPA, or 1-5mM NAM. (A) At all culture time points, trypan blue negative cells were counted. Data represent the fold expansion of megakaryocyte precursors during development. (B) At day 7 and 11, the percentage of apoptotic cells was determined by FACS. Data represent the percentage of Annexin V-positive cells, compared to the control. (C) Absolute numbers of CD61/CD42b positive megakaryocytes were calculated after 11 days of differentiation. Data are representative for 4 independent experiments. Error bars represent SEM, * p<0.05, ** p< 0.01.

To further evaluate the effects of VPA and NAM treatment on megakaryocyte development, we analysed the effects on megakaryocyte (MK) progenitor expansion and survival. Upon treatment of VPA, and NAM, a concentration dependent inhibition of megakaryocyte progenitor expansion was observed as determined by counting trypan blue-negative cells during differentiation (Fig 3A). Next, the effects of VPA, and NAM treatment on MK-progenitor survival were investigated by FACS analysis of early apoptosis markers (Annexin V). While we observed a moderate increase in the percentage of apoptotic cells at day 7 for NAM, this was not observed at day 11 (Fig 3B), suggesting that the effects of VPA and NAM treatment on progenitor expansion are at least not fully caused by increased apoptosis. In agreement with these data, analysis of the absolute megakaryocyte numbers, demonstrated that treatment with increasing concentrations of both VPA and NAM, resulted in a significant decrease in absolute megakaryocyte numbers (Fig 3C). Taken together, these data suggest that VPA treatment inhibits megakaryocyte differentiation and proliferation, while NAM stimulates megakaryocyte differentiation, at the expense of proliferation, in combination with a small relative increase in apoptosis of immature precursors.

### VPA treatment increases the absolute number of MEP

**Fig 4:**
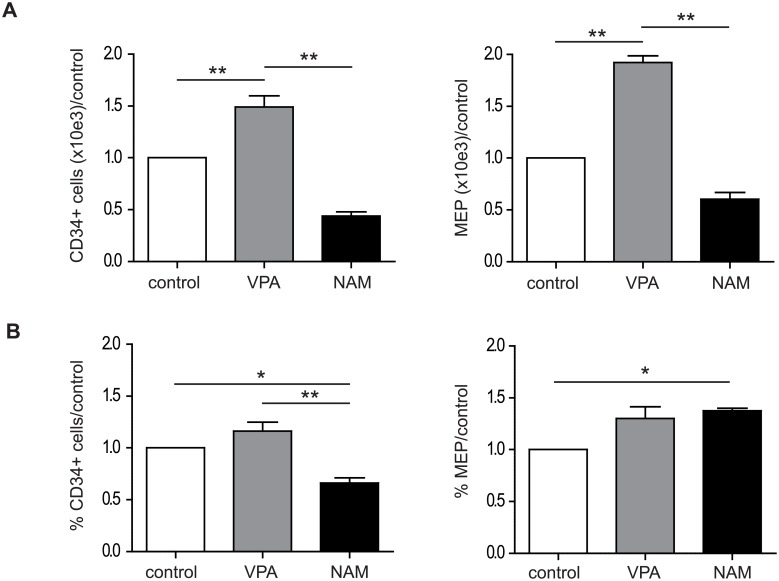
VPA treatment increases the absolute number of MEP. UCB-derived CD34+ cells were differentiated towards megakaryocytes for 4 days in the absence or presence of 200μM VPA, or 5mM NAM. Myeloid progenitor staining was performed according to Manz *et al*. (46), and distinct progenitor populations were analysed by FACS. MEP were gated from the Lin- CD34+, CD38+ CD123- and CD45RA- cell population. Data represent (A) the absolute numbers of CD34+ cells and MEP, compared to the control, and (B) the percentage of CD34+ cells and MEP, compared to the control. Data are representative for 3 independent experiments. Error bars represent SEM, * p<0.05, ** p< 0.01.

We have previously demonstrated that VPA treatment of CD34+ myeloid progenitors, differentiated towards the GM-lineage, resulted in an increased percentage of MEP [51]. To investigate the differential effects of VPA and NAM on MK-progenitors in more detail, we differentiated UCB-derived CD34+ cells towards the MK-lineage for 4 days, and performed FACS analysis of progenitor subsets according to Manz *et al*. [46]. Compared to the control, and NAM treatment, VPA treatment resulted in a significant increase in the absolute number of CD34+ cells, and MEP (Fig 4A), suggesting that VPA treatment stimulates the expansion of MEP. NAM treatment resulted in a significant increase in the percentage of MEP (Fig 4B), while the absolute number was decreased, suggesting that NAM treatment stimulates differentiation towards the ME-lineage, at the expense of progenitor expansion.

In addition to increased expression, and activity of megakaryocyte lineage-specific genes, megakaryocyte development is also dependent on downregulation of erythroid lineage specific genes [52,53]. Since our data suggest that VPA treatment stimulates the expansion of MEP, while MK-differentiation is inhibited, we next investigated the effects of VPA and NAM treatment on erythrocyte progenitor expansion and differentiation. UCB-derived CD34+ cells were isolated, and differentiated to erythrocytes for 11 days. Erythrocyte differentiation was determined based on the expression of the transferrin receptor 1 (CD71), and glycophorin (CD235a), as well as cytospin analysis of erythrocyte morphology. Treatment with 100μM VPA, and 1mM NAM resulted in a significant increase in the expansion of erythrocyte progenitor cells (S2A Fig), which was not observed upon treatment with increasing concentrations, suggesting that VPA and NAM have a concentration-dependent effect on erythrocyte progenitor expansion. Interestingly, while we observed no significant effects of VPA and NAM treatment on CD71/CD235a expression (S2B Fig), cytospin analysis revealed an increase in reticulocytes, illustrated by enucleation (S2C Fig), suggesting that treatment with VPA, and NAM both potentially stimulates erythroid development.

### KDACi treatment increases H3K27 promoter acetylation of ME-lineage specific genes

While VPA and NAM have both been described to affect lysine deacetylase activity, treatment of CD34+ cells resulted in differential effects on MEP expansion and differentiation towards the megakaryocyte differentiation, but the effects on erythroid differentiation were similar. Therefore, we next investigated the potential underlying molecular mechanisms involved in these effects, by differentiating UCB-derived CD34+ cells to megakaryocytes for 4 days, followed by overnight treatment with VPA or NAM, before performing H3K27ac chromatin immunoprecipitation (ChIP). H3K27 acetylation is a histone modification implicated in chromatin remodelling, and characterizes active enhancers and promoters [54]. H3K27ac ChIP, followed by DNA sequencing (ChIPseq) was performed and genes with differentially acetylated promoters or adjacent regions were selected.

**Fig 5:**
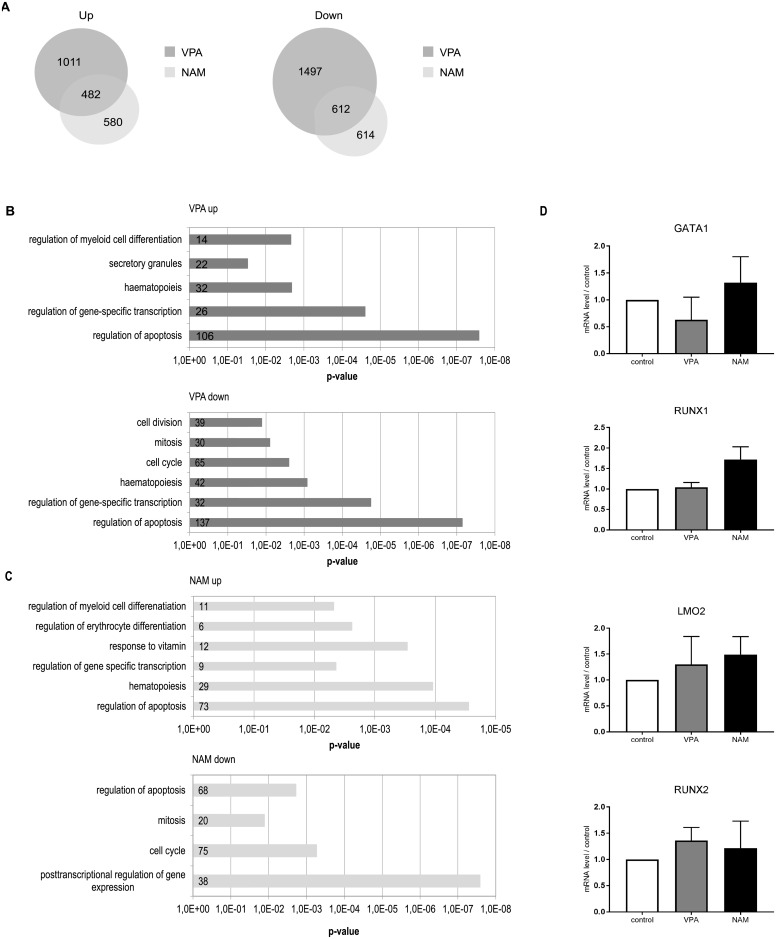
VPA and NAM treatment modulates H3K27 acetylation at the promoters of myeloid specific genes. UCB-derived CD34+ cells were differentiated towards megakaryocytes. At day 4 of differentiation, cells were treated overnight with 200μM, or 5mM NAM. Next, lysates were prepared, followed by ChIP-sequencing (see Methods). Data represent a Venn diagram comparing the number of genes identified based on a >2-fold increase or decrease in H3K27 acetylation levels compared to the control (A). Gene ontology analysis of up- and down-regulated genes by VPA (B) and NAM (C) treatment. From the same cells, RNA was isolated, followed by cDNA synthesis and qPCR (see Methods). Data represent the relative mRNA level of GATA1, RUNX1, LMO2 and RUNX2 compared to the control (D). Data are representative for 3 experiments. Error bars represent SEM, * p<0.05, ** p<0.01.

Treatment with VPA or NAM resulted in 1011 VPA-specific, and 580 NAM-specific genes with > 2-fold increased acetylation, and an overlap of 482 genes. Likewise, we found 1497 VPA-specific, and 614 NAM-specific genes with > 2-fold decreased acetylation, and an overlap of 612 genes (Fig 5A). As expected, because of the restricted nuclear localisation and more general biological functions, inhibition of class I/II HDACs by VPA, resulted in a more dramatic effect on H3K27 acetylation, in both directions (S3 Fig).

Next, utilizing DAVID functional annotation software, gene-ontology analysis was performed. While no specific regulatory network involved in megakaryocyte development was identified, both treatment with VPA and NAM was linked to haematopoiesis and myelopoiesis, apoptosis, and gene-specific transcription (Fig 5B and 5C). Based on further analysis of peak intensity, and promoter proximity, we selected candidate genes with a known functional role in HSC differentiation, myeloid development more generally, or megakaryocyte/erythrocyte development specifically. Treatment with VPA was associated with significant upregulation of AcH3K27 at the megakaryocyte specific genes LIM domain only 2 (LMO2), GA binding protein transcription factor α (GABPA), as well as MYB, a key-regulator of erythroid development, and inhibitor of megakaryocyte development (S4A Fig). Treatment with both VPA, and NAM increased H3K27ac levels at the megakaryocyte-related genes MEIS1, CD36 (thrombospondin receptor), and integrin α 4 (ITGA4, α 4 subunit of VLA4 receptor), (S4B Fig), while NAM treatment specifically increased H3K27ac levels at genes related to HSC function and maintenance, including transcription factor 4 (TCF4), and the megakaryocyte-related gene RAB27b (S4C Fig). Interestingly, treatment with VPA was associated with significantly lower H3K27ac levels at the myeloid specific genes GATA2, as well as RUNX1, a key-regulator of myelopoiesis, and megakaryocyte differentiation specifically (S4D Fig).

Taken together these data demonstrate that VPA and NAM treatment differentially modulates histone acetylation marks, associated with active transcription at genes involved in cell fate decisions during myeloid development, and more specifically ME-lineage differentiation.

### KDACi treatment moderately increases mRNA expression of ME-lineage specific genes

Finally, to investigate whether the changes in H3K27 promotor acetylation of ME-lineage specific genes, indeed lead to increased or decreased mRNA expression, we evaluated the mRNA level of several ME-lineage specific genes, by differentiating UCB-derived CD34+ cells to megakaryocytes for 4 days, followed by overnight treatment with VPA or NAM, before performing real time qRT-PCR. Treatment with VPA resulted in increased mRNA expression of LMO2 and RUNX2, and decreased mRNA expression of GATA1 compared to the control (Fig 5D). Upon treatment with NAM, mRNA expression of GATA1, RUNX1 and LMO2 was increased compared to the control (Fig 5E). This is in line with our data from ChIPseq analysis, demonstrating that treatment with VPA and NAM not only modulates H3K27 acetylation, but indeed influences actual transcription of ME-lineage specific genes.

## Discussion

For the treatment of haematological malignancies, KDACi, in combination with conventional chemotherapy, or other epigenetic agents, have shown to improve haematological outcome [20–27]. Since KDACi are particularly effective in myelodysplastic syndrome (MDS) and acute myeloid leukemia (AML), this suggests that the regulation of protein acetylation is involved in aberrant cellular differentiation in (myeloid) malignancies, as well as normal myeloid differentiation. Treatment with KDACi has been associated with prolonged thrombocytopenia in patients [55], yet the underlying pathophysiological mechanisms are unclear. Recently, it has been demonstrated that treatment with the pan-KDACi abexinostat is associated with p53-dependent apoptosis of megakaryocyte progenitors, and inhibition of the formation of pro-platelets [56]. A study in mice treated with the pan-KDACi panbinostat suggested that thrombocytopenia is the result of defective platelet release for megakaryocytes, while megakaryocyte progenitor development and survival was not affected [57]. In this study, utilising an *ex-vivo* human CD34+ differentiation system, we investigated the effects of VPA, a widely used class I, and to a lesser extent, class IIa KDACi [58, 59], and the SIRT1/2 inhibitor NAM on normal megakaryocyte development. VPA treatment stimulated expansion of MEP, while terminal differentiation was inhibited (Figs 2 and 4), suggesting that class I (and IIa) HDAC activity is important for normal megakaryocyte development. Wilting *et al*. demonstrated that the combined conditional deletion of HDAC1 and HDAC2, both class I HDAC, in human haematopoietic cells, resulted in anaemia and thrombocytopenia, accompanied by apoptosis of megakaryocytes, while single deletions resulted in no haematological phenotype. In addition it was suggested that high HDAC1 expression levels are required for ME-lineage development, while HDAC1 overexpression has been demonstrated in AML cell lines, and primary AML cells [60, 61]. Studies utilising the myeloid cell line K562 have demonstrated that VPA treatment induces proteosomal degradation of HDAC2 specifically, and inhibits erythroid differentiation [36, 59]. Taken together, this suggests that the activity of HDAC1, or HDAC2 (based on functional redundancy) is required for megakaryocyte and erythrocyte development, and that these HDAC play a role in normal, and aberrant myeloid differentiation [62]. Taken into account that class II HDACs are predominantly expressed in non-haematological tissues [63], our data suggest that the effects of VPA treatment on megakaryocyte development are the result of HDAC1/2 inhibition predominantly. In contrast with these studies, we observed no effects on erythroid development upon treatment with VPA, suggesting that the remaining levels of HDAC1 (and HDAC2) are sufficient for erythroid development, or that VPA treatment results in increased activity of other HDAC, including HDAC9, which has been demonstrated in AML cells, and has been functionally linked to erythroid development, as well as the pathogenesis of myeloproliferative disorders [64, 65].

ChIP-sequencing analysis demonstrated that VPA treatment of megakaryocyte progenitors was associated with increased acetylation of H3K27 at the promoters of genes involved in HSC differentiation, and ME-lineage development (Fig 5 and S4 Fig). H3K27ac is associated with active transcription, suggesting that genes marked with this epigenetic modification are being actively transcribed [54]. These include LMO2, a gene involved in both megakaryocyte, and erythrocyte differentiation, and MYB, a lineage-directing gene for erythroid development. Combined with the effects of VPA treatment on GATA-2 (reduced H3K27ac signal), and MEIS1 (increased H3K27ac signal), involved in MEP formation, this could explain the effects we observed upon VPA treatment on the expansion of MEP, and disrupted megakaryocyte lineage development [7, 52, 66–69]. We confirmed the effect on active transcription of several of these genes (Fig 5E).

Treatment with the SIRT1/2 inhibitor NAM resulted in accelerated differentiation, and increased polyploidisation of megakaryocyte precursors at the expense of proliferation (Figs 2 and 4). Little is known concerning the role of sirtuins in normal haematopoiesis, and investigations focusing on SIRT1 have been hampered by high perinatal mortality of SIRT1 knockout mice [70–72]. In addition to the effects of SIRT1 on histone deacetylation, the functional effects of SIRT1 predominantly involve deacetylation of non-histone proteins, including p53, and the forkhead family of transcription factors (FOXO) [73, 74], implicating a role in a large variety of biological processes, including stress responses, and control of lifespan, including for HSC [75]. Our data suggest that SIRT1/2 inhibition accelerates megakaryocyte development, and thereby reduces the lifespan of megakaryocyte precursors. Normal megakaryocyte development includes three successive steps: proliferation, polyploidisation, and cytoplasmic maturation, involving distinct transcriptional regulators [6, 76]. While it has been previously demonstrated that SIRT1 inhibition stimulates polyploidisation, the underlying molecular mechanisms responsible for these effects in the context of ME-lineage development, and megakaryocyte differentiation, are still unclear [42, 77]. Both from our ChIP-sequencing data, as from our qPCR data (Fig 5 and S4 Fig), we can conclude that upon treatment with NAM, there is upregulation of RUNX1, a transcription factor most active in terminal megakaryocyte differentiation. Furthermore, we have compared our ChIP-sequencing data with existing microarray based profiles derived from distinct stages of megakaryocyte development, and ploidy levels [78, 79]. In these studies, polyploidisation, and differentiation was accompanied by upregulation of RAB27b, a member of the Rab family of small GTPases. RAB27b is a downstream effector of Fli-1 and NF-E2, which was modulated by NAM treatment in the current study (S4 Fig), and has been implicated in (pro) platelet formation [80, 81]. In addition, megakaryocyte differentiation was associated with decreased expression of the histone-lysine N-methyltransferase Enhancer of Zeste Homologue 2 (EZH2) [79]. Interestingly, both VPA, and NAM treatment were associated with loss of promoter AcH3H27 of EZH2 (data not shown), a Polycomb-group protein, regulating methylation of H3K27, and subsequent transcriptional repression. EZH2 activity is important for HSC function in normal, and aberrant myelopoiesis, and has been linked to early stage megakaryocyte development [79–83]. Evaluation of the regulatory effects of SIRT inhibition on AcH3K27-mediated transcriptional activity requires further investigation. Moreover, since NAM treatment resulted in dramatic effects on megakaryocyte development, which can be only be partially explained by our ChIP sequencing data, this suggests that SIRT inhibition predominantly affects non-histone substrates. Preliminary data in megakaryocyte precursor cells, suggest that NAM treatment activates FOXO3a, a well described SIRT1 target protein, involved in megakaryocyte proliferation, and survival (data not shown) [84].

Possibly, patients with thrombocytopenia of unknown origin could benefit from NAM treatment, as it might increase megakaryocyte differentiation and therefore peripheral blood platelet counts. On the opposite, due to possible skewing of ME-lineage differentiation toward the erythroid lineage, in patients with (non-haemolytic) anaemia, VPA might increase the red blood cell count and haemoglobin levels.

In conclusion, we have demonstrated that VPA and NAM, two functionally distinct KDACi, differentially regulate cell fate decisions during megakaryocyte, and erythroid development. Our data suggest that these effects can at least be partially explained by direct effects on H3K27-promoter acetylation, and subsequent transcription of genes involved in HSC and haematopoietic progenitor differentiation, and more specifically in ME-lineage development. Taken together our data increase the understanding of the effects of KDACi on normal haematopoiesis, and the role of HDAC and sirtuins in the regulation of normal myeloid development and megakaryocyte development in particular.

## Supporting information

S1 Fig Schematic overview of megakaryocyte development. HSC differentiate into the bipotent MEP, followed by megakaryocyte lineage progression, characterized by polyploidization, and cytoplasmic maturation, accompanied by sequential increased surface expression of integrin ß3 (CD61), and glycoprotein Ib (CD42b), and activity of the transcription factors GATA1, RUNX1, GATA2, LMO2, FOG1, NF-E2, FLI1 and TAL1.
(EPS)

S2 Fig VPA and NAM have concentration dependent effects on erythroid development. UCB-derived CD34+ cells were differentiated to erythrocytes during 11 days in the absence or presence of 100–200μM, or 1-5mM NAM. (A) At all culture time points, trypan blue negative cells were counted. Data represent the fold expansion of erythroid precursors during development. (B) At day 11 of differentiation, surface marker expression of CD71, and CD235a was analysed by FACS. Data represent the percentage of double positive cells, compared to the control. (C) Cytospin analysis of erythroid precursor cells. Arrows indicate enucleation. Data are representative for 3 (VPA), or 2 (NAM) independent experiments. Error bars represent SEM, * p<0.05, ** p< 0.01.
(EPS)

S3 Fig VPA treatment results in more genes being differentially acetylated at H3K27 compared to NAM treatment. UCB-derived CD34+ cells were differentiated towards megakaryocytes. At day 4 of differentiation, cells were treated overnight with 200μM, or 5mM NAM. Next, lysates were prepared, followed by ChIP-sequencing (see Methods). Data represent the relative number of genes with a >2-fold increase (A) or decrease (B) of H3K27 acetylation compared to the control.
(EPS)

S4 Fig VPA and NAM treatment influences H3K27 acetylation at genes implicated in HSC function, and ME-lineage development. UCB-derived CD34+ cells were differentiated towards megakaryocytes. At day 4 of differentiation, cells were treated overnight with 200μM VPA, or 5mM NAM. Next, lysates were prepared, followed by ChIP-sequencing (see Methods). Plots represent H3K27ac signal in the vicinity of LMO2, GABPA, MYB (A), MEIS1, CD36, ITGA4 (B), TCF4, RAB27b (C), GATA2 and RUNX1 (D) gene loci.
(EPS)
